# Epstein-Barr virus myelitis and Castleman's disease in a patient with acquired immune deficiency syndrome: a case report

**DOI:** 10.1186/1752-1947-5-209

**Published:** 2011-05-27

**Authors:** Costantine Albany, George Psevdos, Jasminka Balderacchi, Victoria L Sharp

**Affiliations:** 1 Department of Medicine, St Luke's-Roosevelt Hospital Center, New York, NY, USA; 2 Division of Infectious Disease, St Luke's-Roosevelt Columbia University College of Physicians and Surgeons, New York, NY, USA; 3 Department of Pathology, St Luke's-Roosevelt Hospital Center, New York, NY, USA; 4 Center of Comprehensive Care, St Luke's-Roosevelt Hospital, 1000 10th Ave, Suite 2T, New York, NY, 10019, USA

## Abstract

**Introduction:**

Few cases of Epstein-Barr virus myelitis have been described in the literature. Multi-centric Castleman's disease is a lymphoproliferative disorder that is well known for its associations with the human immunodeficiency virus, human herpes virus 8, and Kaposi's sarcoma. The concurrent presentation of these two diseases in a patient at the same time is extremely unusual.

**Case Presentation:**

We describe the case of a 43-year-old Caucasian man with acquired immune deficiency syndrome who presented with fever, weight loss and diffuse lymphadenopathy, and was diagnosed with multi-centric Castleman's disease. He presented three weeks later with lower extremity weakness and urinary retention, at which time cerebrospinal fluid contained lymphocytic pleocytosis and elevated protein. Magnetic resonance imaging demonstrated abnormal spinal cord signal intensity over several cervical and thoracic segments, suggesting the diagnosis of myelitis. Our patient was ultimately diagnosed with Epstein-Barr virus myelitis, as Epstein-Barr virus DNA was detected by polymerase chain reaction in the cerebrospinal fluid.

**Conclusion:**

To the best of our knowledge, this is the first case of multi-centric Castleman's disease followed by acute Epstein-Barr virus myelitis in a human immunodeficiency virus-infected patient. Clinicians caring for human immunodeficiency virus-infected patients should be vigilant about monitoring patients with increasing lymphadenopathy, prompting thorough diagnostic investigations when necessary.

## Introduction

The Ebstein-Barr virus (EBV) is a member of the herpesviridae family. Even though most infections with EBV go unnoticed, in some cases it can be associated with the development of potentially serious conditions, including infectious mononucleosis. EBV has been associated with central nervous system (CNS) diseases (encephalitis and meningitis), but rarely as a cause of myelitis [1,2]. The pathogenesis of EBV-associated CNS disorders is not completely understood. While acute EBV myelitis is rare, its occurrence in patients who are infected with human immunodeficiency virus (HIV) or who have acquired immunodeficiency syndrome (AIDS) is even more uncommon.

Multi-centric Castleman's disease (MCD) is a systemic disorder characterized by significant peripheral lymphadenopathy and hepatosplenomegaly, as well as frequent fevers, night sweats, fatigue and weight loss [3]. HIV-infected individuals appear to be at an increased risk for MCD [4]. MCD also is well known to have an association with human herpes virus 8 (HHV-8) and Kaposi's sarcoma (KS) [5]. Although no standard of care has been established, MCD is often treated with aggressive systemic immunomodulatory therapy [6].

## Case Presentation

A 43-year-old Caucasian man on anti-retroviral therapy (lopinavir/ritonavi and emtricitabine/tenofovir) for HIV/AIDS presented in September 2008 with a three-month history of low grade fevers, night sweats, generalized fatigue, lethargy, unintentional weight loss, and bilateral lower extremity swelling. He had a CD4 count of 196 cells/mm^3^, and a viral load of <50 copies/mL. He had been diagnosed with HIV-infection in 1990; his CD4 nadir was 4 (0.6%) in February 2004 with high viremia, 175,671 copies/mL. After initiation of potent anti-retroviral therapy the HIV viral load has been undetectable since October 2004.

A physical exam of our patient revealed diffuse lymphadenopathy in his cervical, axillary and inguinal areas, splenomegaly, and pitting edema in both lower extremities. Computed tomography of his chest, abdomen and pelvis demonstrated widespread mediastinal, hilar, axillary, retro-peritoneal and pelvic lymphadenopathy, as well as the splenomegaly. A laboratory workup revealed a hemoglobin level of 8.5 mg/dL, a white blood cell count (WBC) of 4,600 cells/*μ*L, an albumin level of 2.5 mg/dL, and a Westergren sedimentation rate of 90 mm/hr. A lower extremity Doppler was negative for deep venous thrombosis.

**Figure 1:**
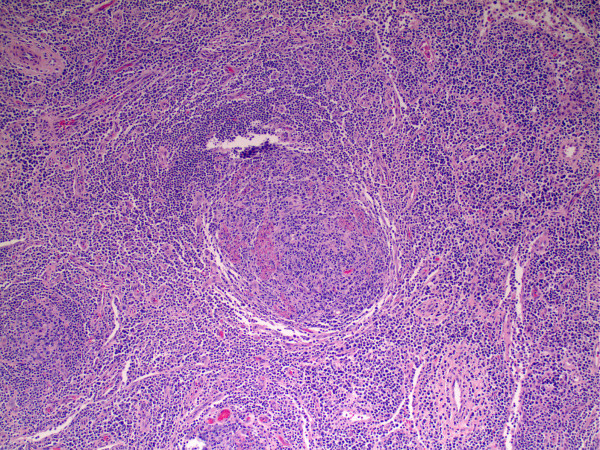
**Histological specimen of enlarged lymph node stained with hematoxylin and eosin**. Reveals marked plasma cell infiltration, and follicles that were variable in appearance, from marked follicular hyperplasia to involution and dendritic cell hyperplasia.

**Figure 2:**
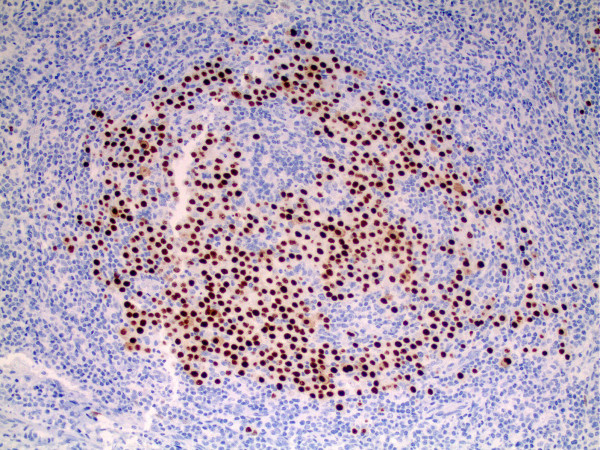
**Immunohistochemical study demonstrates HHV-8 positive**.

Histological examination of lymph node biopsy material revealed marked plasma cell infiltration, and follicles that were variable in appearance, from marked follicular hyperplasia to involution and dendritic cell hyperplasia (Figure 1). Immunohistochemical studies demonstrated HHV-8 positive (Figure 2), and plasmablastic foci associated with an intense polytypic plasma cell infiltrate. These findings were consistent with the diagnosis of MCD--plasma cell variant. Our patient was discharged from the hospital and was scheduled to start treatment for MCD.

Three weeks later our patient presented with lower abdominal pain, urinary retention, and lower extremity weakness. Neurological examination revealed bilaterally reduced motor power (4/5) in all major muscle groups in his lower extremities, along with a positive Babinski sign on the right. Deep tendon reflexes were normal bilaterally. An ultrasound of his pelvis demonstrated bladder distention. A Foley catheter was inserted and 2 liters of urine was evacuated, with complete relief of pain.

**Figure 3:**
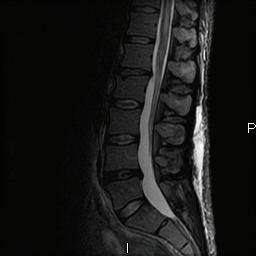
**Thoracic-lumbar spine MRI with gadolinium enhancement**. There is increased signal in the spinal cord and lumbosacral roots on sagittal view.

A Gadolinium magnetic resonance imaging (MRI) of his spine was done to rule out spinal cord compression; it revealed abnormal spinal cord signal intensity involving several cervical and thoracic segments, associated with expansion of the cord and mild enhancement of the areas of abnormal T2 signal (Figure 3). MRI of the brain revealed a few patchy areas of abnormal T2 signal in the peri-ventricular and pontine white matter.

A lumbar puncture was performed: cerebrospinal fluid (CSF) contained an elevated WBC of 50 cells/mm^3^, with 90% lymphocytes, and a high protein level of 243 mg/dL; cytological analysis identified mature reactive lymphocytes with no evidence of lymphoma on flow cytometry. Polymerase chain reaction (PCR) was positive for EBV DNA in the CSF, but negative for HHV-8, cytomegalovirus (CMV), herpes simplex virus (HSV) and Varicella-Zoster virus (VZV). Treatment with high-dose dexamethasone was initiated. Our patient was then transferred to the National Institute of Health (NIH) and started on a treatment protocol of high-dose zidovudine (AZT) and valganciclovir for MCD (ClinicalTrials.gov identifier: NCT00099073). Subsequent laboratory follow-up revealed marked improvement over the first seven days of treatment, including a marked decrease in WBC and protein in his CSF. A follow-up MRI demonstrated resolution of all spinal cord lesions. He is now doing well, his last CD4 was 322 (15%), viral load < 50 copies/mL in August 2010.

## Discussion

This case is notable for two reasons. First, a 43-year-old man with AIDS initially suspected to have lymphoma due to his clinical features at presentation, was subsequently diagnosed with MCD. Second, within three weeks he presented with EBV myelitis.

With respect to the former condition, this case brings to light the importance of obtaining definitive histological diagnoses in HIV-infected patients who present with lymphadenopathy and systemic symptoms. MCD is relatively uncommon cause for such a presentation. Though clinically similar to lymphoma, MCD is an entity that is distinct from malignant lymphoproliferative disorders in both histology and prognosis. It is characterized by significant peripheral lymphadenopathy and hepatosplenomegaly, as well as by frequent fevers, night sweats, fatigue and weight loss [7]. Abnormal laboratory findings include pancytopenia, elevated liver function tests, raised C-reactive protein and interleukin-6 (IL-6), and hypergammaglobulinemia [3,8]. The condition typically presents in patients between the ages of 50 and 65 years, but HIV-infected patients tend to be younger at presentation, as in our case, and can be at an increased risk for MCD [4,9]. This disease often arises concurrently with KS. It has been reported that, among HIV-positive patients with MCD who are infected with HHV-8, up to 70 percent will develop KS at some time during their clinical course. MCD may present at any CD4 count in these patients [4].

Although no standard treatment has been established, MCD often is treated systemically [6] with such aggressive remission-induction chemotherapy regimens as cyclophosphamide-doxorubicin-vincristine-prednisone (CHOP) and doxorubicin-bleomycin-vincristine; immunomodulatory agents like thalidomide and interferon alpha; and monoclonal antibodies, like those directed against the IL-6 receptor (atlizumab) and CD20 (rituximab) [10,11]. The contribution of aggressive anti-retroviral treatment to the treatment of MCD remains controversial. Novel treatments targeted at HHV-8 have yielded promising results. Our patient was enrolled in an NIH clinical trial of high-dose oral zidovudine and valganciclovir for MCD ClinicalTrials.gov identifier: NCT00099073.

EBV is a γ-herpes virus that is detectable in over 90 percent of the general population. Even though most infections with EBV go unnoticed, in some cases it can be associated with the development of serious conditions, including infectious mononucleosis. EBV has been associated with CNS diseases, including meningitis and encephalitis [1,2]. EBV myelitis is uncommon, but is important in the differential diagnosis of acute myelopathy. Acute EBV myelitis can present as neurologic dysfunction due to involvement of the white matter. When EBV infection affects only part of the transverse expanse of the spinal cord, it manifests as asymmetric motor and sensory symptoms. The pathogenesis of EBV-associated CNS disorders is not completely understood, but may be due to direct viral invasion of the CNS. Alternatively, damage may be immunologically-mediated via the infiltration of cytotoxic CD8+ lymphocytes into neural tissue, or the deposition of antibody-antigen complexes [1]. EBV infection may cause mild symptoms of mononucleosis, like pharyngitis, prior to the onset of acute myelitis [12].

Patients with AIDS have 10 to 20 times as many circulating EBV-infected B-cells as those who are healthy. T-cells from patients with AIDS suppress EBV-infected B-cells less effectively than do cells from normal controls [13]. A decline in EBV-specific cytotoxic T-cells and an elevated EBV viral load precedes the development of EBV-associated non-Hodgkin's lymphomas in HIV-infected patients; however, these changes are not seen in patients with HIV before the development of opportunistic infections. HIV viral load and the progression of HIV disease do not appear to be affected by primary infection with EBV [14].

In our patient, both the clinical and radiological findings were characteristic of myeloradiculopathy. The CSF abnormalities further indicated that the disease was inflammatory. Extensive virological analysis of serum and CSF revealed the presence of EBV DNA in the CSF, as well as the absence of CMV, HSV, VZV and HHV-8. The clinical features of myelitis, and the increased signal in the cervical and thoracic spinal cord in this patient on MRI, were similar to those described in previously reported EBV myelitis cases [2,15-17].

The International Herpes Management Forum recommends that the diagnosis of EBV infections of the CNS can include evidence of EBV DNA in CSF by PCR. Unfortunately, there is no definitive treatment for EBV infection of the nervous system. Steroids and immunoglobulin have been used empirically, but their effects on disease progression are unknown. Anti-viral therapy has not demonstrated clinical efficacy in the treatment of EBV-related CNS disorders [1].

## Conclusions

Our patient presented with two rare disorders in rapid succession, first MCD, followed by EBV myelitis. Histological evaluation revealed the first diagnosis, and CSF PCR analysis the second. Clinicians caring for HIV-infected patients should be vigilant about monitoring patients with increasing lymphadenopathy, prompting thorough diagnostic investigations when necessary. Treatment for MCD can be complex, and referral to dedicated programs with unique treatment protocols should be considered. EBV myelitis is too rare for there to be published therapeutic trials; nonetheless, systemic steroids may be beneficial in its treatment.

## Abbreviations

AIDS: acquired immunodeficiency syndrome; CNS: central nervous system; CSF: cerebrospinal fluid; CMV: cytomegalovirus: EBV: Ebstein-Barr virus; HHV-8: human herpes virus 8; HSV: human simplex virus; KS: Kaposi's sarcoma; MCD: multi-centric Castleman's disease; MRI: magnetic resonance imaging; PCR: polymerase chain reaction; VZV: Varicella-Zoster virus; WBC: white blood cell count.

## Consent

Written informed consent was obtained from the patient for publication of this case report and any accompanying images. A copy of the written consent is available for review by the Editor-in-Chief of this journal.

## Competing interests

The authors declare that they have no competing interests.

## Authors' contributions

CA, GP and VS analyzed and interpreted the patient data and wrote the manuscript. JB performed the histopathologic and immunohistochemical analyses of the biopsy, and was a major contributor in writing the manuscript. All authors read and approved the final manuscript.
